# Feedback control of upright seating with functional neuromuscular stimulation during a reaching task after spinal cord injury: a feasibility study

**DOI:** 10.1186/s12984-022-01113-4

**Published:** 2022-12-12

**Authors:** Aidan R. W. Friederich, Xuefeng Bao, Ronald J. Triolo, Musa L. Audu

**Affiliations:** 1 Department of Biomedical Engineering, Case Western Reserve University, OH Cleveland, USA; 2 Advanced Technology Center, Louis Stokes Veterans Affairs Hospital, OH Cleveland, USA

**Keywords:** Feedback control, Functional neuromuscular stimulation, Neuroprosthesis, Seated stability, Spinal cord injury, Musculoskeletal

## Abstract

**Background:**

Restoring or improving seated stability after spinal cord injury (SCI) can improve the ability to perform activities of daily living by providing a dynamic, yet stable, base for upper extremity motion. Seated stability can be obtained with activation of the otherwise paralyzed trunk and hip musculature with neural stimulation, which has been shown to extend upper limb reach and improve seated posture.

**Methods:**

We implemented a proportional, integral, derivative (PID) controller to maintain upright seated posture by simultaneously modulating both forward flexion and lateral bending with functional neuromuscular stimulation. The controller was tested with a functional reaching task meant to require trunk movements and impart internal perturbations through rapid changes in inertia due to acquiring, moving, and replacing objects with one upper extremity. Five subjects with SCI at various injury levels who had received implanted stimulators targeting their trunk and hip muscles participated in the study. Each subject was asked to move a weighted jar radially from a center home station to one of three target stations. The task was performed with the controller active, inactive, or with a constant low level of neural stimulation. Trunk pitch (flexion) and roll (lateral bending) angles were measured with motion capture and plotted against each other to generate elliptical movement profiles for each task and condition. Postural sway was quantified by calculating the ellipse area. Additionally, the mean effective reach (distance between the shoulder and wrist) and the time required to return to an upright posture was determined during reaching movements.

**Results:**

Postural sway was reduced by the controller in two of the subjects, and mean effective reach was increased in three subjects and decreased for one. Analysis of the major direction of motion showed return to upright movements were quickened by 0.17 to 0.32 s. A 15 to 25% improvement over low/no stimulation was observed for four subjects.

**Conclusion:**

These results suggest that feedback control of neural stimulation is a viable way to maintain upright seated posture by facilitating trunk movements necessary to complete reaching tasks in individuals with SCI. Replication of these findings on a larger number of subjects would be necessary for generalization to the various segments of the SCI population.

## Background

Spinal cord injury (SCI) decreases overall quality of life due to a plethora of potential adverse effects to many body systems that compromise musculoskeletal, urinary, cardiovascular, and other essential functions. Quality of life is heavily reliant on the level of physical function, with individuals capable of a higher level of independence experiencing a higher quality of life [1, 2]. Health preference scores are a measure of health-related quality of life and have been reported as 0.93 for the general population [3], 0.58 for stroke survivors [4], and 0.42 for those with Parkinson’s disease [5]. Because SCI severely compromises motor and sensory function below the level of injury this population has one of the lowest health preference scores of any disability group at 0.27 [3]. It is critical to improve the intrinsic function of individuals with SCI as even small increases in their physical capabilities will have drastic effects on quality of life and independence.

An under-examined aspect of functional independence is the role of trunk stability in facilitating activities of daily living (ADLs) by providing support against destabilizing perturbations while using the upper extremities. Trunk movement enables ADLs by enlarging the workspace [6] and are even employed when an object or ADL is within arm’s length [6, 7]. For these reasons, trunk stability has been consistently rated as a high priority for improvement after SCI [8–10]. Trunk stability is also an important factor for preventing falls in individuals with SCI [11] and can promote balance and stable function in the wheelchair [12].

Functional neuromuscular stimulation (FNS) has successfully provided options for people with SCI to independently perform many activities affected by paralysis, including reaching [13], biking [14], and walking [15]. Applying a constant level of stimulation to the nerves serving the hip and trunk muscles improves seated posture during quiet sitting [16] and increases reaching distances [17]. However, stimulation needs to be modulated appropriately to compensate for different loading scenarios and applied perturbations that could destabilize seated posture. Perturbations can arise from both external (e.g., wheelchair collisions) and internal (e.g., manipulating heavy objects) sources. Various forms of feedback controllers have been proposed to maintain seated balance in the sagittal plane by adjusting stimulation parameters to resist external perturbations applied to the trunk [18, 19]. Murphy et al. [20] and Bheemreddy et al. [21] implemented a threshold-based self-righting controller to recruit trunk muscles with supramaximal stimulation once the subject exceeded a predetermined trunk flexion or bending threshold to effectively return the user to an erect posture from large forward flexed or sideways bending postures. Proportional, integral, derivative (PID) control was found to be particularly appropriate for upright sitting since the extrinsic (active muscles) properties of the trunk are dominated by the proportional and derivative model components [22]. The goal of this study was to expand these previous control architectures to maintain stability in the presence of internal perturbations generated during both sagittal and coronal plane movements while performing functional reaching tasks simulating typical ADLs. The intended effect of the PID control law was to increase levels of stimulation whenever the trunk moved away from the baseline erect position, thus returning the subject to erect after the internal perturbations and minimizing the resultant overall postural sway.

The objectives of this study were to: (1) implement a multi-directional controller to maintain upright seated posture, and (2) determine the effects of the controller on posture during a functional reaching task. We hypothesized that the controller would reduce the postural sway during a functional reaching task and will quicken return to erect movements compared to without stimulation or applying low levels of stimulation to ensure a baseline constant stiffness of hips and trunk.

## Methods

### Participants and technology

Five individuals (S1, S2, . . ., S5) with SCI at varying thoracic and cervical levels participated in this study. Each participant had previously received an implanted neuroprosthesis for other studies intended to restore standing, walking, or postural balance. The neuroprosthesis is composed of a stimulator-telemeter [23, 24] connected to intramuscular, epimysial, or nerve cuff electrodes surgically placed to activate nerves serving the muscle groups spanning the trunk and hips. Each subject completed reconditioning exercises at home with the system for several months before the experiments. Table 1 lists the anthropometric and neurological characteristics of each subject at the time of testing, along with the muscle groups targeted by the controller. Supplemental surface stimulation was used in two of the subjects (S3 and S5) to augment the actions of the implanted system. The specific muscle groups activated with surface stimulation are marked with an asterisk (*) in Table 1. Subject S5 had a complete injury at T10 and only a small section of his trunk was paralyzed. We therefore anticipated that the controller’s effect would be reduced on him compared to those with higher injury levels and less volitional trunk control. All subjects were informed of all aspects of the experiment and subsequently signed consent forms approved by the local institutional review board (IRB: VA Northeast Ohio Healthcare System, Protocol Number: 07101-H36, Approval Date: 9/7/2010 or IRB: VA Northeast Ohio Healthcare System, Protocol Number: 18037-H20, Approval Date: 11/2/2018).

**Table 1:** Clinical characteristics of study participants and the muscles activated. Muscles shown were available for activation bilaterally

Subject	Age (y)	Gender	Height (cm)	Weight (kg)	Injury level	AIS$^{+}$ grade	Injury date	Implant date	Stimulated muscle groups
S1	50	M	175.3	82	C5	C	12/9/2014	10/23/2019	ES, QL, IL, HS, GX, GM
S2	50	F	167.6	58.5	C7	B	3/12/1998	11/12/1999	ES, QL, PA, HS, GX, GM
S3	56	M	172.7	71.7	T3	A	3/30/2013	12/2/2014	ES, QL*, IL, PA, HS, GX, GM
S4	46	F	172.7	84.9	T4	A	2/13/2012	10/20/2014	ES, QL, IL, PA, GX
S5	32	M	182.9	74.8	T10	A	10/19/2007	12/12/2018	ES*, QL*, IL, GX, HS, PA

*ES* lumbar erector spinae, *QL* quadratus lumborum, *PA* posterior portion of adductor magnus, *GX* gluteus maximus, *GM* gluteus medius, *IL* iliopsoas, *HS* hamstring semimembranosus

$^{+}$American Spinal Injury Association Impairment Score (AIS)

*Activated with surface stimulation

### Feedback control system

**Fig. 1:**
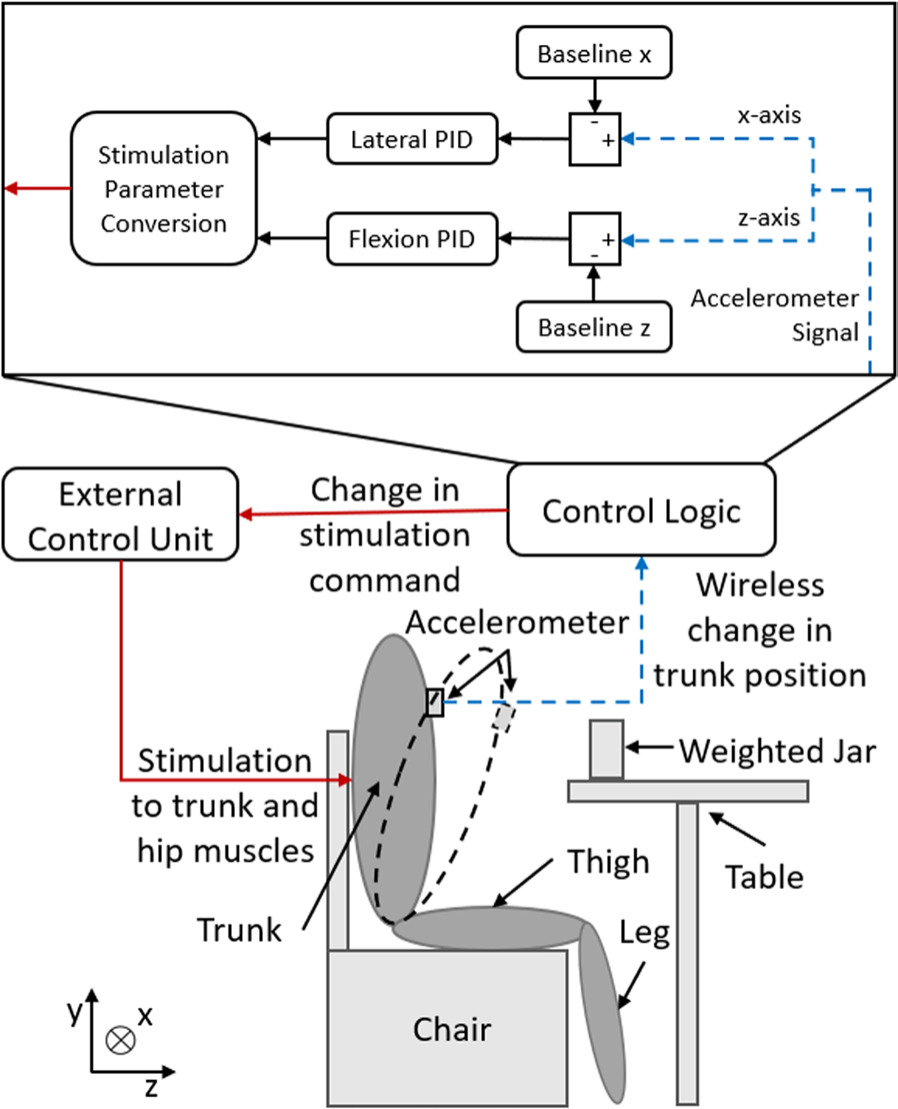
Experimental setup and block diagram of the control system. The subject was seated in front of a table with a weighted jar. The dashed black oval shows the trunk during a flexion movement. Trunk position was obtained from an accelerometer and compared to baseline values from quiet upright seating. The two PID controllers determined the necessary changes in stimulation parameters that the external control unit issued to the implanted neuroprosthesis

The feedback control system (Fig. 1) was composed of a small wireless tri-axial accelerometer, PID control law, and an external control unit to communicate with the implanted neuroprosthesis. The 3-axis accelerometer (CMA3000- D01, VTI Technologies, Vantaa, Finland) was placed on the sternum aligned approximately with the body anatomic axes and its output signal was sampled at 40 Hz. The sensor predominately measured trunk tilt using variation in the component of acceleration due to gravity along one of its axes. As the subject leaned away from the erect position the gravity vector projection on the local accelerometer coordinate frame changed such that variation in the x-axis value corresponded to lateral bending of the trunk, while changes in the z-axis (Fig. 1) corresponded to trunk flexion/extension [20, 21, 25]. Baseline x and z values were set at the beginning of each trial while the subject assumed an upright seated posture. Deviation from baseline was routed to independent PID controllers for sagittal and coronal pane movements governed by Eq. 1 [19, 26].

1 $$\begin{aligned} {\textstyle C(t_k) = P \cdot e(t_k) + I \cdot \frac{T_s}{e(t_k)-e(t_{k-1})} + D \cdot \frac{N}{1+N \cdot \frac{T_s}{e(t_k)-e(t_{k-1})}}} \end{aligned}$$

where C(t$_{\textrm{k}}$) is the controller gain and the variables P, I, D are the proportional, integral, and derivative terms respectively, T$_{\textrm{s}}$ is the sample time (0.025 s), and N is the derivative filter coefficient and refers to the bandwidth of the lowpass filter applied to the derivative term (N=100 for all experiments). The error at each time point (e(t$_{\textrm{k}}$)), was determined by subtracting the baseline x and z acceleration values from the measured accelerometer values. Equation 1 defined the control laws for both flexion/extension and lateral bending movements. During flexion movements all muscle groups on both sides of the body were activated (lumbar erector spinae, quadratus lumborum, posterior portion of adductor magnus, gluteus maximus, gluteus medius, semimembranosus). The iliopsoas was only activated if the trunk extended past the baseline z acceleration value. Surface stimulation of the rectus abdominus was also considered, however in initial testing activation of this muscle did not cause significant flexion movement from an upright position. Additionally, our group has found that activation of the abdominals can be uncomfortable and cause breathing difficulties [27]. In practical use, the wheelchair’s backrest will provide support against backward perturbations. During lateral bending movements only the erector spinae (ES) and quadratus lumborum (QL) opposite to the direction of movement were activated. For example, if the subject leaned left, only the right ES and QL were activated to control the movement. Stimulation parameters sent to the external control unit to activate the subject’s muscles were determined by Equation 2.

2 $$\begin{aligned} {\textstyle p^i_{applied} = p^i_{min} + C(t_k)\cdot (p^i_{max} - p^i_{min})} \end{aligned}$$

Where p$^{i}_{\textrm{applied}}$ is the pulse width (PW) applied to each stimulating channel i of the neuroprosthesis, p$^{i}_{\textrm{max}}$ is the maximum allowable PW of each channel i determined by increasing stimulation until it became uncomfortable for the subject or hardware limits were reached (250 µs), and p$^{i}_{\textrm{min}}$ is the minimum PW similarly determined as the lowest value at which visible movement was produced by the target muscle. Stimulation amplitude was kept at a constant 20 mA unless the subject reported discomfort, in which case the amplitude was decreased to a more comfortable level. In cases where both flexion and lateral bending occurred, a muscle group was controlled by both controllers causing redundancies. These were resolved by choosing the larger PW value that resulted from the two controllers.

### Experimental setup

**Fig. 2:**
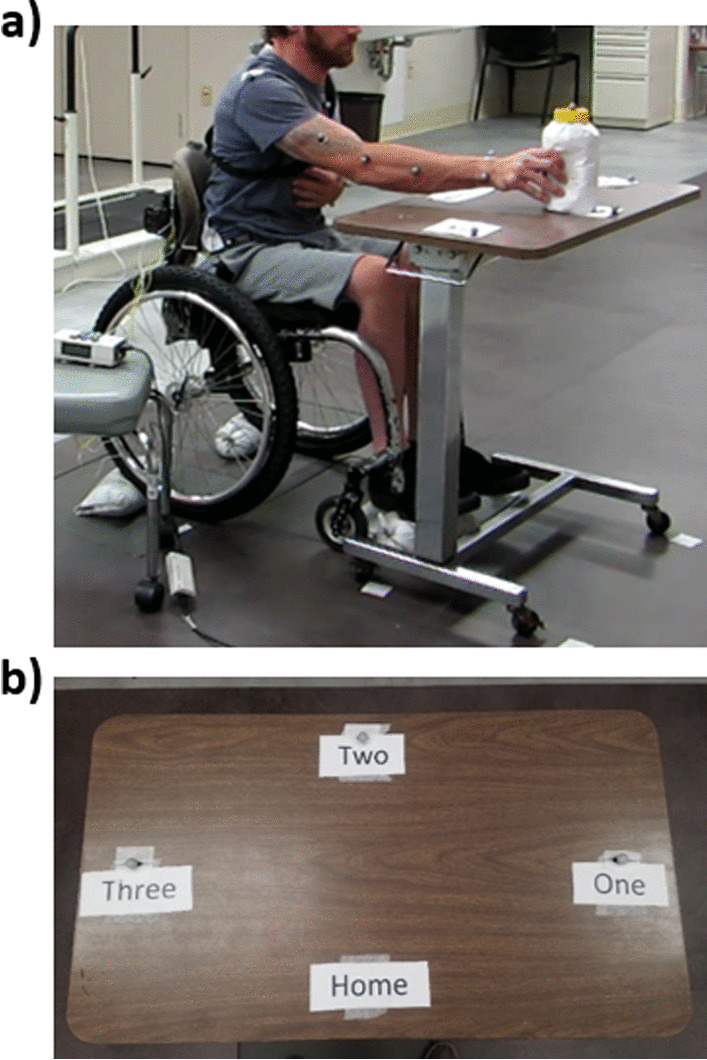
The experiment setup. Subjects remained in their wheelchairs (**a**) in front of a table marked with a ’home’ station and three target stations (**b**)

A weighted jar was placed on a table marked with four stations, a central ’home’ station surrounded by three radially located target stations labeled ’one’, ’two’, and ’three’ (Fig. 2). Each subject completed the task with only their dominant hand. In the remainder of the report, we will refer to the numbered stations as “ipsilateral” (station closest the dominant hand), “midline” (station two), and “contralateral” (station farthest from the dominant hand). Only subject S2 completed the tasks with her left hand, the other subjects used their right hand. The weight of the jar was varied from 0.9 to 2.26 kg depending on estimated shoulder and arm strength and comfort [17]. Kinematics of the trunk movements were obtained with a 16-camera motion capture system sampling at 100 Hz (Vicon Motion Systems Ltd., Oxford, UK). Reflective markers were placed on the C7 vertebrae, sacrum, and bilaterally on the acromion of the scapula, greater trochanter of the femur, middle of the upper arm, the lateral and medial epicondyle of the elbow, middle of the lower arm, the lateral and medial wrists, and the anterior superior iliac spine of the pelvis. Markers were also placed at each of the stations and on the weighted jar.

### Functional task procedure

The prescribed task was designed to reflect typical ADLs that would occur in the home. Thus, subjects sat in their own wheelchairs in front of the testing table (Fig. 2a), with their non-active hand resting unconstrained across their chests or lower abdomens. Participants were allowed to catch themselves with either extremity if they felt a fall could occur due to trunk destabilization. Subjects were asked to move a weighted jar to and from the target stations (Fig. 2b) in predetermined random order. Distance to the table was customized for each individual to ensure that the jar could be transferred to each of the target stations and returned to home without fear of falling. Subjects were instructed to (1) lift the jar from the home station, (2) move it to the selected target station on cue, (3) release the jar, (4) return their empty hand to home, and (5) resume an upright seated position. Upon receipt of a second cue, the subject reached for the jar at the target station, acquired it, and returned it to the home station. The subjects rested between every 9 reaches to reduce the effects of fatigue, and completed as many of these reaching tasks within a single session as possible, depending on the amount of time required for controller tuning and their own personal time constraints. All data were initially included in the analysis. The number of reaches each subject performed under each condition is shown in Table 2. Subject S2 had a low level of constant stimulation applied to aid in completion of the reaching task while the controller was inactive. This was done at her request since she was a regular user of low level constant stimulation to add stiffness to her sitting posture at home on a daily basis.

**Table 2:** Number of reaches performed by each subject under each condition and in total

	Condition	Number of reaches to each target station			
		Ipsilateral	Midline	Contralateral	Total
S1	No stimulation	9	9	9	27
	Feedback control	9	9	9	27
S2	Low constant stimulation	12	12	12	36
	Feedback control	12	12	12	36
S3	No stimulation	15	15	15	45
	Feedback control	15	15	15	45
S4	No stimulation	15	15	15	45
	Feedback control	15	15	15	45
S5	No stimulation	9	9	9	27
	Feedback control	12	12	12	36

### Data analysis, outcome measures, and statistics

**Fig. 3:**
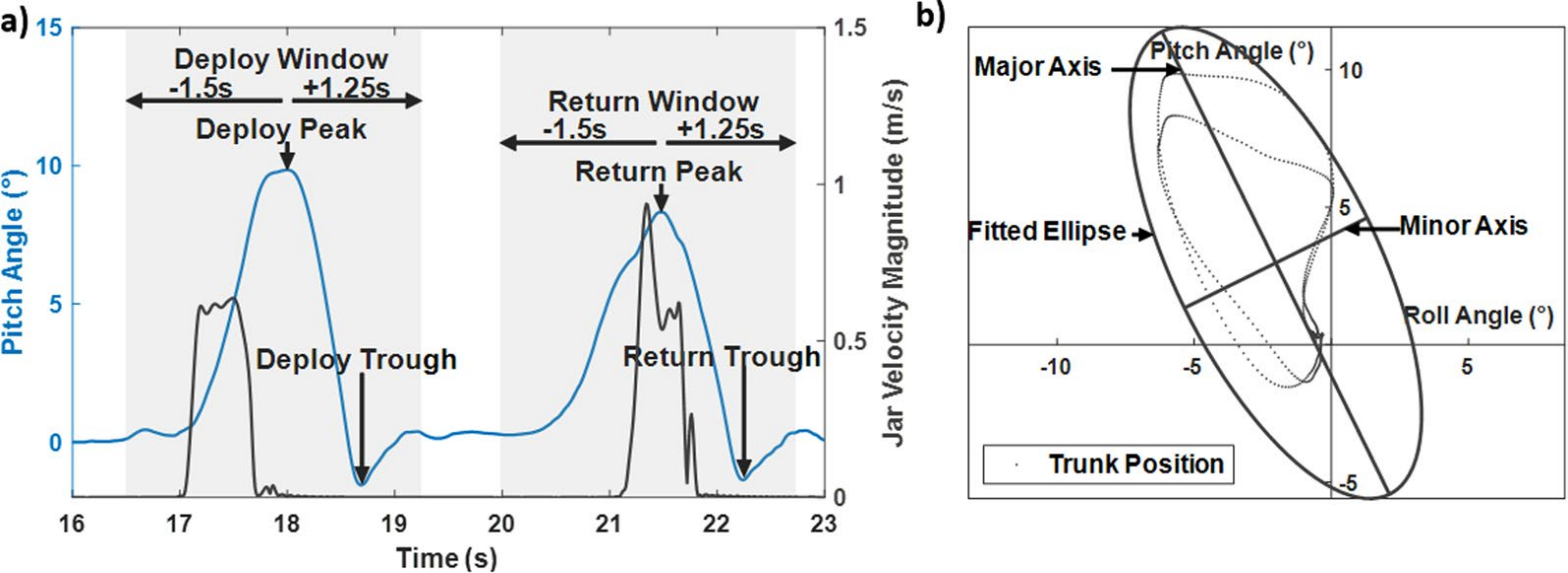
Example of the data from a representative trial. Movements of the jar were determined from the jar velocity to identify deploy and return peaks and troughs. The data were then windowed to 1.5 s before the peak and 1.25 s after the peak to fully capture the movement (**a**). The trunk pitch and roll angle are plotted against each other, and an ellipse is fitted to them. Major and minor axis of movement are determined from the fitted ellipse (**b**)

*Isolating reaching movements:* Trunk pitch (extension and flexion motion) and trunk roll (lateral bending) angles were derived from the motion capture data offline during post-processing with MATLAB 2020 (MathWorks, Natick, MA). These measures differ from the tilt information derived from the mounted accelerometer that was only used for real-time control of the trunk. The trunk pitch and roll angles were determined by the angle between the global reference frame and the line defined between the sacrum and C7 marker [28]. Figure 3a shows the plot from a typical trial and the parameters used to process the data. We identified each of the reaching movements automatically based on when the jar marker reached 10% of its maximum velocity. Once each movement was isolated, we centered the data for analysis around the most significant landmark, the peak pitch angle. The data were windowed to 1.5 s before and 1.25 s after the peak pitch angle for subjects S2, S3, S4, S5. Subject S1 completed the task at a slower pace due to reduced hand function; therefore, his data were windowed to 3 s before and 2.5 s after the peak pitch. This process was applied to both the deployment of the jar to the target station and returning it to the home station. The data were normalized to 100% of the movement cycle with the first 50% defining the deployment and the second 50% the return.

*Prediction ellipse area:* We assessed the trunk kinematics with the area of the ellipse defined by the trunk pitch vs roll angles of each maneuver. The ellipse area (Fig. 3b) quantifies postural sway and represents a subject’s ability to control trunk movements and preserve stability. Larger ellipse areas indicate poorer seated stability [29, 30]. The ellipse area was determined by fitting an ellipse to a plot of the trunk pitch and trunk roll angles with the prediction ellipse method [31]. The prediction ellipse estimates the area in which future observations will occur with 95% probability.

*Mean effective reach:* We also examined the mean effective reach of the active arm relative to the body axis and was defined as the distance between the shoulder and wrist markers. The mean effective reach is a measure of combined shoulder elevation and elbow extension and reflects the ability of the subjects to hold objects further away from the trunk [16].

*Return to upright time:* Return to upright time was determined separately for deployment and return motions. It was calculated as the difference between the peak trunk angle and the following trough defined as the minimum trunk angle directly following the peak (Fig. 3a). Quicker return to upright motions enable the subject to reduce the length of time in a potentially unstable leaning position.

*Statistics:* The no/low constant stimulation condition was compared to the feedback control condition with Wilcoxon rank-sum tests as some of the data were determined to be non-normal based on the Anderson-Darling test. All statistical comparisons of ellipse area, mean effective reach, and return to upright times were performed with a single-subject experimental design where each subject served as their own control.

### Analysis of continuous trunk movement curves

The previous analyses examined specific outcome measures. To explore any additional unanticipated impacts of the controller on trunk movements, we further examined the movement of the trunk along the major axis of movement (identified by the prediction ellipse in section E) according to the methods established by Duhamel et al. [32]. These methods allow comparison of the same population under two conditions (no/low constant stimulation and feedback control stimulation) throughout the entire movement cycle to identify effects not captured with the above outcome measures. This is done by characterizing each subject with a single curve by censoring outlier curves and averaging the remaining. Then, 95% confidence bands determined whether the two conditions were similar throughout the entire movement cycle.

**Fig. 4:**
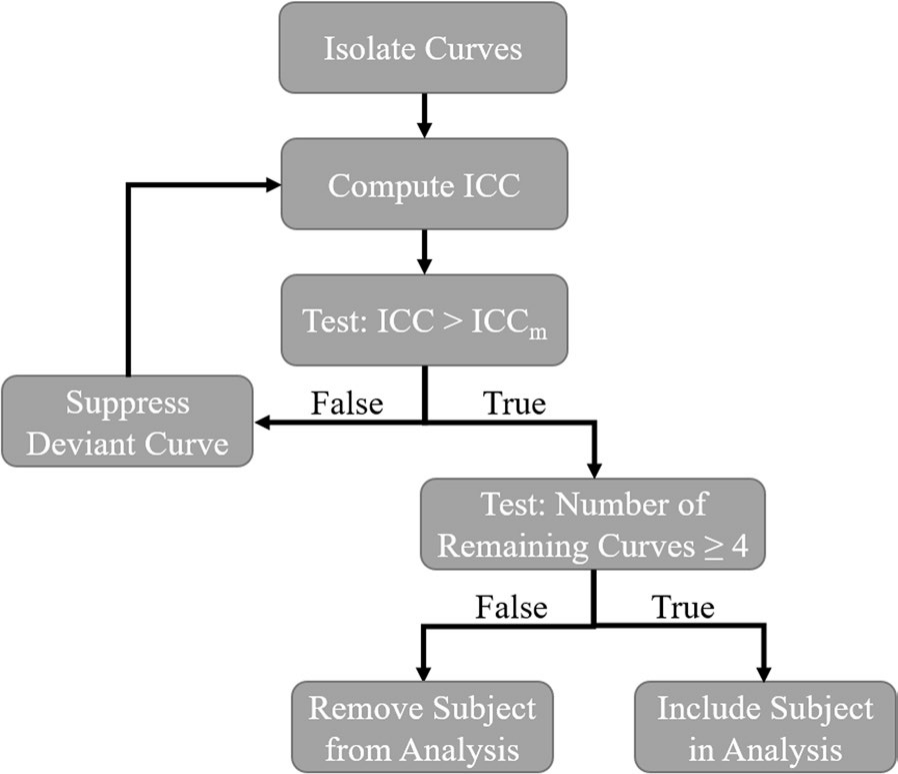
A flowchart showing the process for assessing curve reliability

*Curve analysis on trunk movement:* The goal of defining the trunk movements of each subject by a single curve for each condition was complicated by the high degree of variation observed in the kinematics of individuals with musculoskeletal disorders [33] that could corrupt a typical mean curve. To remove atypical curves before ensemble averaging, we assessed reliability with intraclass correlation coefficients (ICC). ICCs have been shown to account for both differences in means among measures and parallelism between test results [34]. ICC was calculated for each subject during each condition and target station. ICC estimates and their 95% confidence intervals were calculated based on repeated measurements, absolute-agreement, and a 2-way mixed-effects model [35]. To obtain reliable curves for each subject the ICC was calculated for the whole set of measures (subject, stimulation condition, and target station, 369 curves total) and a minimum level ICC$_{\textrm{m}}$ was identified based on the probability density function of the ICCs estimated with a Gaussian kernel. An ICC$_{\textrm{m}}$ = 0.9 was chosen, as this was the main inflection point of the Gaussian kernel plot. Figure 4 shows the process of assessing the trunk movement curves for reliability. First, the curves were grouped based on subject, condition, and target station, similar to Table 2. Second, ICC was computed for each set of curves. Third, if the ICC > ICC$_{\textrm{m}}$, for a given group then all curves were included in subsequent analysis, and if not the least similar curve was removed. The second and third steps were repeated until ICC > ICC$_{\textrm{m}}$ for all remaining curves. If ICC remained below ICC$_{\textrm{m}}$ when only four curves remained in the set, data for that given group was ignored. Each subject was ultimately represented by a single mean curve for each target station taken as the ensemble average of the reliable set of curves.

*Confidence bands* The 95% confidence band for the difference between the means of the two conditions (feedback control vs no/low constant stimulation) was computed with the bootstrap method [32, 36]. All bootstrap bands were constructed using 1000 iterations. This test allowed us to determine when the feedback control had an impact on trunk movements during the reaching tasks.

## Results

### Outcome measures

Table 3 summarizes the statistical results for each outcome measure. Application of feedback controlled stimulation resulted in reduced postural sway for two of the subjects (S1, S4), allowed three subjects to extend their arms farther to accomplish the tasks (S1, S2, S3), and quickened return to upright motion for four subjects (S1, S2, S3, S4).

**Table 3:** Statistical results of each outcome measure for each subject

		Outcome measures			
Subject	Injury level	Ellipse area	Mean effective reach	Upright return time	Upright deploy time
S1	C5	${\varvec{\downarrow }}$	${\varvec{\uparrow }}$	${\varvec{\downarrow }}$	–
S2	C7	–	${\varvec{\uparrow }}$	${\varvec{\downarrow }}$	–
S3	T3	–	${\varvec{\uparrow }}$	${\varvec{\downarrow }}$	–
S4	T4	${\varvec{\downarrow }}$	${\varvec{\downarrow }}$	${\varvec{\downarrow }}$	${\varvec{\downarrow }}$
S5	T10	–	–	–	–

${\uparrow }$ indicates the outcome measure statistically increased when feedback modulated stimulation was applied. ${\downarrow }$ indicates the outcome measure statistically decreased and – indicates no significant was found

**Fig. 5:**
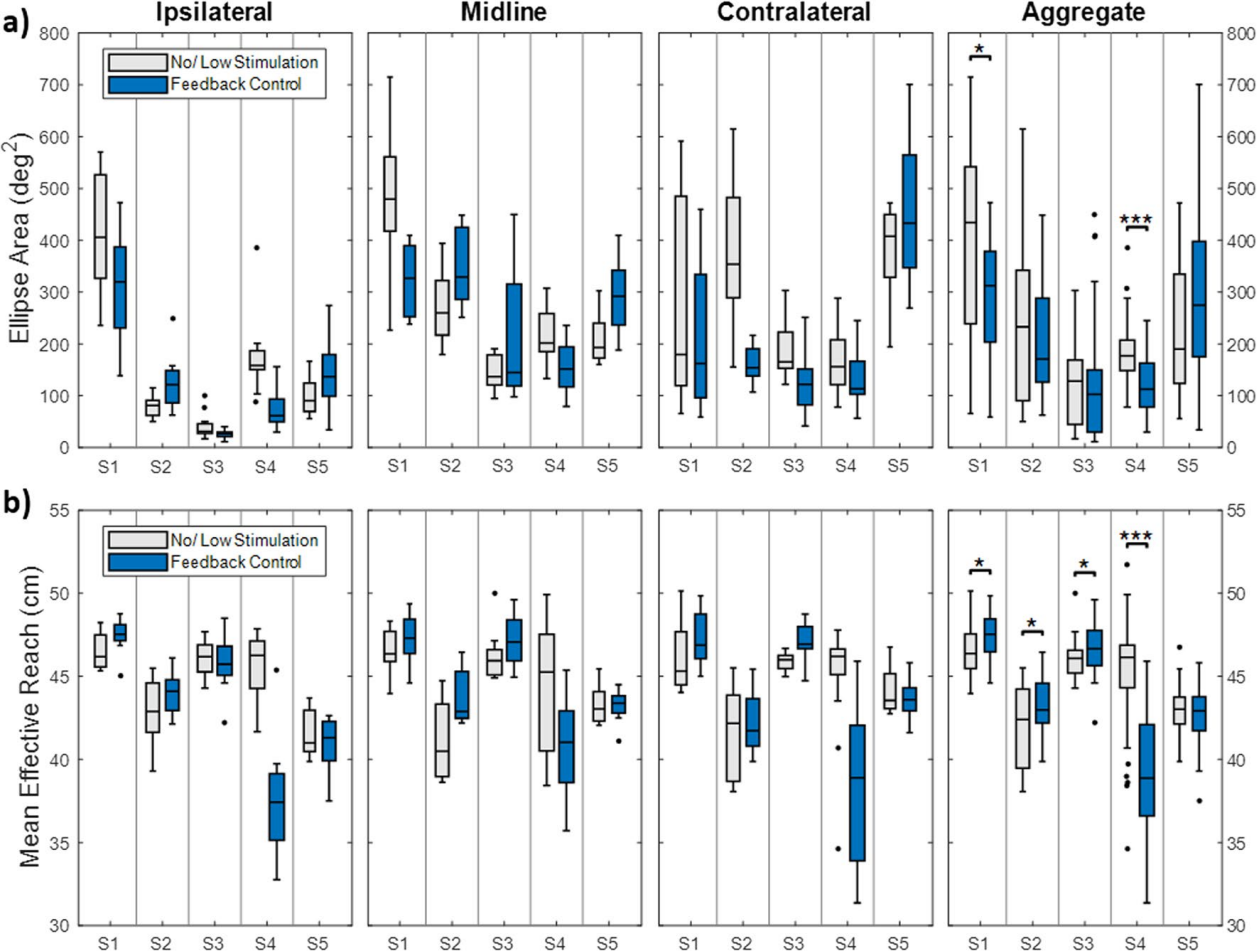
Ellipse area of trunk pitch and roll angle (**a**) and mean effective reach (**b**) of each subject during reaching movements to the ipsilateral, midline, and contralateral stations. Black dots indicate outliers. Aggregate data is shown in the last column. Statistics were performed on aggregate data only. *p < 0.05, **p < 0.01, ***p < 0.001

*Prediction ellipse area:* Ellipse area is shown in Fig. 5a. The results are separated into each target station followed by the aggregate data. Statistics were only performed on the aggregate data. Generally, ellipse areas were lowest for the ipsilateral station and greater for the midline and contralateral stations. There was a statistically significant reduction of ellipse area with feedback control for subjects S1 (z = 2.28, p = 0.022) and S4 (z = 4.69, p < 0.001) only.

*Mean effective reach:* The mean effective reach (Fig. 5b) did not show any consistent trends between the three stations. Statistically significant increases were observed for subjects S1 (z = − 2.14, p = 0.032), S2 (z = − 2.68, p = 0.007), and S3 (z = − 2.47, p = 0.014). A statistically significant decrease was observed for S4 (z = 6.19, p < 0.001). No differences were observed for subject S5.

**Fig. 6:**
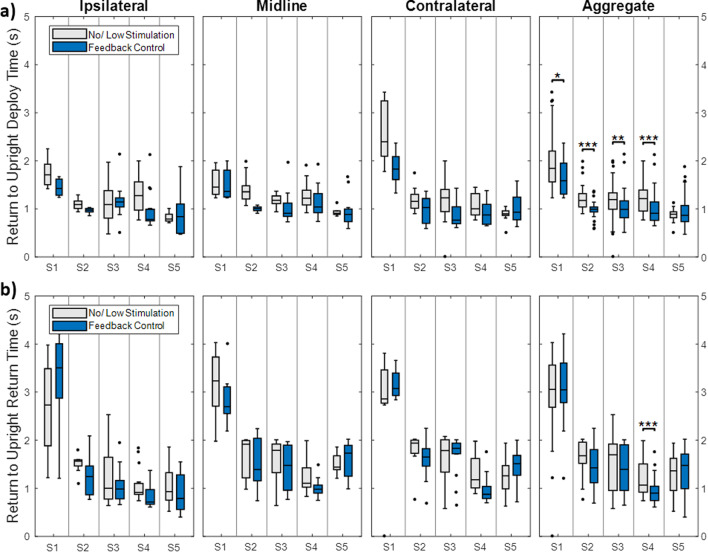
The time required for the subject to return to an upright position after deploying the jar to an away station (**a**) and returning the jar to the home station (**b**). Data are separated into the ipsilateral, midline, and contralateral stations and aggregate data is shown in the last column. Statistics were performed on aggregate data only. *p < 0.05, **p < 0.01, ***p < 0.001

*Return to upright time:* The time required for the subject to return to an upright position generally varied from 0.5 to 2 seconds, with S1 taking up to 4 seconds (Fig. 6). The return to upright time after deploying the jar to any target station was statistically faster with the controller active for subjects S1 (0.32s faster, z = 2.11, p = 0.035), S2 (0.18 s faster, z = 4.6, p < 0.001), S3 (0.21 s faster, z = 2.89, p = 0.004), and S4 (0.18 s faster, z = 3.72, p < 0.001) (Fig. 6a). Only S4 saw a significant time to upright reduction when returning the jar home (0.17 s faster, z = 4.02, p < 0.001) (Fig. 6b).

### Analysis of continuous trunk movement curves

*Censuring deviant curves:* The trunk movement curves from subject S3 to the ipsilateral station were considered unreliable from the ICC analysis. No subject curves were considered unreliable for the midline station, and the movements of subject S1 were considered unreliable for the contralateral station.

**Fig. 7:**
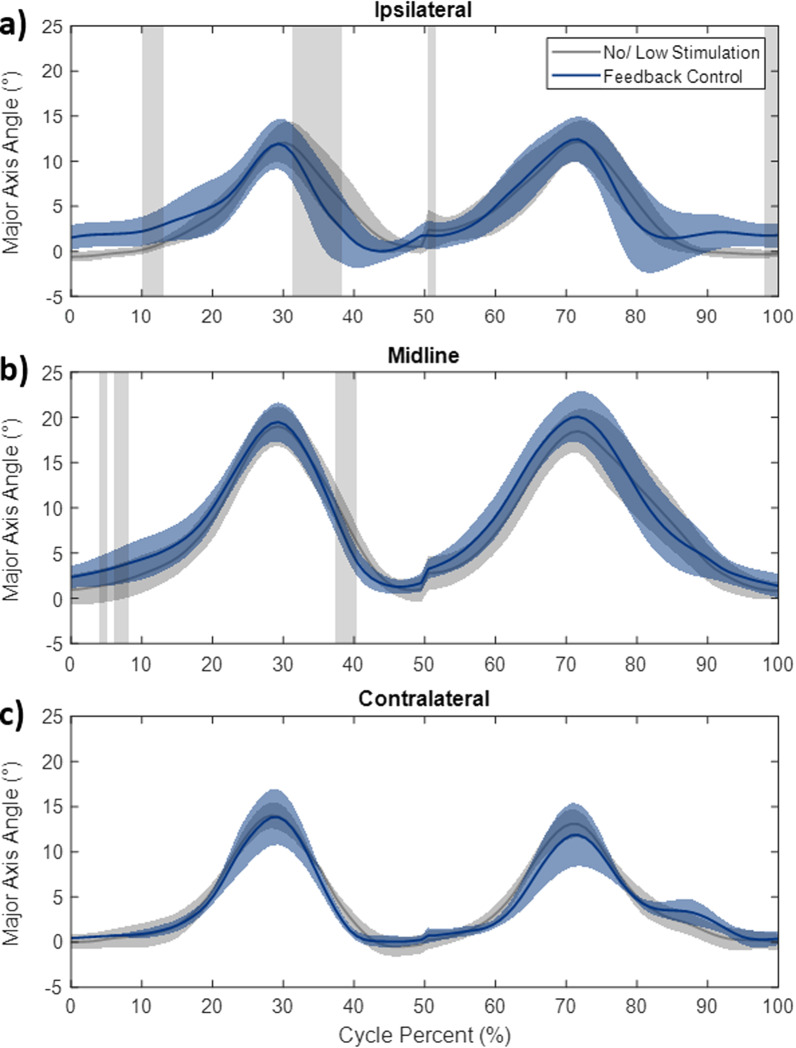
Trunk angle along the major action of movement plotted against the cycle percent for ipsilateral (**a**), midline (**b**), and contralateral (**c**) stations. Solid lines indicate the mean angle of all subjects and shaded colored areas are standard deviation. Horizontal grey shaded areas show a significant difference between the no/low stimulation and feedback control conditions determined with the Bootstrap analysis

*Curve analysis on trunk movement:* Figure 7 shows the movement of the trunk along the major axis during both conditions after removal of the unreliable curves. Horizontal areas shaded in grey show where the bootstrap test determined there was a significant difference between the two conditions. These differences occurred both in trunk movements during ipsilateral and midline stations with no differences observed in the contralateral station. The differences are concentrated in two areas: the return to erect motion and the starting posture. The feedback controller resulted in a quickened return to upright motion after deploying the jar to the ipsilateral and midline position. Figure 7a shows average trunk movement curves as a result of reaching to the ipsilateral station. While the peak excursion occurs at 29 cycle percent for both conditions a significant difference occurred between 31 and 38 percent cycle during the return to upright motion. Feedback control resulted in shallower trunk angles at the same point in the cycle and showed a return to upright at 41 percent cycle compared with feedback control to 48 cycle percent with no/low stimulation. A similar effect can be observed during the other return to erect motions; however, this was not found to be significant during reaching to the contralateral station or during motions returning the jar to the home station. The feedback control also resulted in increases in the starting angle of the subjects during these two motions.

## Discussion

Maintaining a stable base of support is necessary to complete many ADLs, as they often impart internal perturbations as a consequence of moving objects from one location to another. Upper extremity actions, including driving, eating, and dressing all impart unique destabilizing disturbances on the trunk, requiring an equal and opposite reaction to counteract the effect of the disturbances. We have demonstrated a feedback control system capable of maintaining an upright posture in response to reaching tasks by simultaneously controlling trunk extension and lateral bending in individuals with paralysis.

Ellipse area, while typically utilized to analyze posturography during quiet sitting [31, 37], is applicable to understanding the range of trunk movement our subjects employed to accomplish the reaching task [38]. We initially hypothesized that feedback control would reduce the ellipse area, as larger movements away from erect results in increased levels of stimulation based on the control logic. Subjects S1 and S4 both showed a significantly reduced ellipse area, supporting this hypothesis. A non-significant reduction was observed in S2 and S3 and a trending, but non-significant increase was observed for S5. The compensatory strategies employed by those subjects likely influenced this outcome. For example, while every subject was asked to place their non-active hand on their stomach and only employ it in the case they sensed an impending fall, the non-active arm was sometimes used to press the hand on the abdomen. This may have aided in stiffening the trunk by increasing intra-abdominal pressure which has been associated with increases in spinal stability [39, 40], much like wearing an abdominal belt has been shown to increase lumbar stiffness [41]. It is possible pressing on their abdomen achieved a similar result.

Three subjects (S1, S2, S3) saw significant increases in mean effective unilateral reach distance with feedback control. This indicates these subjects chose to or were able to hold the weighted jar farther away from their center of mass when the controller was active, which is consistent with the effects of constant levels of FNS applied to trunk muscles during bimanual reach [16, 17]. In contrast, S4 experienced a significant decrease in mean effective reach as well as a decrease in ellipse area or overall trunk movement with the controller. During the feedback control condition, S4 immediately withdrew her arm to her chest after releasing the jar to the target stations, while during the no stimulation case she commonly placed her arm on her thigh to catch herself in instances of instability, resulting in a lower mean effective reach despite decreased trunk movements when the controller was active.

A major benefit of the feedback control is improved return to upright motion. The controller quickened the return to upright motion of four subjects during deployment of the jar to the target stations. Improvement ranged from 0.17 to 0.32s, a decrease of between 15 and 25%, compared to the no/low stimulation case. While the difference is small for a single reaching movement, implementation of feedback control at home can quicken return to upright every time the trunk is away from erect. Individuals with SCI typically seek to maintain an upright posture while reaching by employing strategies that provide counterbalance to the reaching arm such as extension of the trunk [33]. Returning the subject to an upright stable posture faster after a reach will reduce the length of time in an unstable posture. In instances with possible falls it is also crucial to restore upright sitting as fast as possible [21, 42]. The return to upright movements are likely a combination of eccentric and concentric contractions. Eccentric contractions of hip and trunk extensor muscles occur when the controller activates during deployment of the trunk, as those muscles would be lengthening. On the other hand, when the subject is returning to upright, the contractions of those muscles become concentric. This organization possibly benefits the return to upright motion as the stronger eccentric contractions serve to counter the initial momentum of the trunk during deployment, while the concentric contractions return the user upright.

Prior experiments have demonstrated the ability of a threshold-based controller to return a person to an upright position after a large trunk disturbance [20, 21]. These benefits continued to be evident during smaller internal disturbances caused by an upright reaching task in the current study. Interestingly, the return to upright motion was minimally affected by stimulation when the subject returned the jar to the home station. One possible explanation is due to the subjects pushing down on the table with their upper extremity through the jar, thereby propelling their trunk to the upright position without need for stimulation assistance. This compensatory strategy has been reported before during bimanual reaching tasks [33, 43] and was observed in all our subjects. It was especially prevalent in subject S5, who has a complete injury at the T10 level and as such retains more voluntary control of his trunk musculature, which may be why the controller had limited effects on his movement. This suggests that it may be reasonable to expect that a neuroprosthesis for seated reaching or postural balance will have greatest benefit to recipients with higher levels of injury and least control of their hip and trunk musculature. A similar reliance on injury level was also reported while assessing the effects of constant stimulation on hip and trunk muscles during various functional activities [17].

Previous work on FNS of the trunk has focused on bimanual tasks [16, 17], thus eliminating the majority of compensatory strategies available to individuals with complete SCI. Here we attempted to create a realistic unimanual task by having subjects in their own wheelchairs and allowing them to catch themselves in instances of instability. This resulted in compensatory strategies including increased hand pressure on the abdomen, pressing down on the jar to return the trunk to erect, and placing the active arm on their thigh to prevent forward trunk pitch. The full impact of a neuroprosthesis for trunk stability on reducing or eliminating reliance on such compensatory measures remains to be investigated. In other disability conditions where there is impaired control of the upper limbs, such as in stroke survivors [44, 45] and upper limb prosthesis users [46], exaggerated trunk movements are commonly utilized and required to perform reaching tasks. Facilitating trunk movements that complement the desired reaching motions would likely change or possibly reduce the compensatory strategies exhibited by the subjects in this study.

While a qualitative study, the participants did express a preference for reaching with the controller active. Perturbation resistance is important in many activities of daily living. Our subjects have expressed interest in utilizing improved trunk stability for everyday tasks, such as driving, returning to upright after acquiring items, and food preparation and cleanup. Improved seated stability has been correlated with improved completion of a variety of upper-body tasks, including dressing [47], typing, eating, moving boxes, opening doors, etc. [48]. Designing a controller that is both resistant to perturbations and capable of holding upright or leaning postures could improve seated stability thus aiding in these activities of daily living.

There are several limitations of this study. First, the controller was tested with five subjects and showed encouraging, though varied effects. The control system should be applied to a larger group of users with different presentations before the results can be generalized to the entire population. Second, trunk axial rotation (twist) was ignored and only trunk flexion and lateral bending were addressed. Currently, control of axial rotation was not achievable with the muscle groups being activated by our neuroprosthesis and would require targeting additional trunk muscles such as the internal and external obliques. Incorporating axial rotation may require attention in the future especially for reaching of objects directly to the side of the subject. Third, muscles were assigned to a given motion (flexion, extension or lateral bending) and stimulated based on the same control signal regardless of their specific recruitment properties. Future control schemes should seek to identify and incorporate subject-specific muscle synergies and recruitment properties. Fourth, the feedback signal for control was obtained from raw voltage signals of one accelerometer placed on the sternum. An improved measure of trunk position could be obtained with orientation data from a 9-axis inertial measurement unit (IMU) or a network of sensors combined to yield a high fidelity estimate of trunk orientation. Additionally, because the neuromusculoskeletal system is nonlinear, non-autonomous, and partially unobservable, the linear PID control law is only valid for a limited operating range of the trunk and pelvis [49]. In the upright seated position, the proportional and derivative terms dominate the extrinsic trunk properties [22], suggesting that a PID controller is valid during quiet sitting. Here we may have exceeded the linear operating range with trunk excursions of over 20° (Fig. 7b and c). This is a possible explanation of why the improvement in the return to erect motion was most evident in the closer ipsilateral station movements and less prevalent during reaching toward the midline and contralateral stations that required greater trunk excursion (Fig. 7). Future work should explore nonlinear control schemes, such as a model predictive controller [50] or a linear controller in combination with knowledge of the subject specific nonlinear muscle properties. Finally, it is possible that despite best efforts, fatigue may have accumulated within testing sessions and impacted results. We minimized the effects by resting between each trial and ensuring that each subject completed recondition exercises at home with the system for several months before the experiments.

Future work should also focus on translating the controller out of the laboratory and into the user’s home. The Networked Neuroprosthesis is a promising target for integrating these controllers in everyday life due to containing the necessary electrodes, implanted accelerometers and processing capabilities [51]. Through a home-going trial we can directly examine the effects of feedback controlled trunk movements on the user’s quality of life and the direct effects on activities of daily living.

## Conclusion

It is imperative to improve quality of life for those with an SCI. Increasing one’s functional capabilities will directly benefit their ability to perform ADLs, thus improving quality of life. The work presented here supports the potential for feedback control of stimulation to ensure upright sitting to have an impact on simulated ADLs that cause internal perturbations, thereby resulting in instability to seated balance for persons with SCI. Feedback-controlled functional neuromuscular stimulation aided in returning individuals with low cervical to low thoracic SCI to an upright seated position after performing unimanual reaching tasks that involved manipulating weighted objects. We hypothesized that the control scheme would reduce overall postural sway and quicken return to upright trunk motion. These hypotheses were supported in two and four of the subjects, respectively, with return to upright being most robust. Although our hypotheses were generally supported, the variation in individual outcomes indicates that the study should be replicated in a larger sample of the SCI population. Completion of the unimanual task was often accomplished with aid from compensatory strategies. Future design of home going controllers should be aware of the effects of compensatory strategies and either target reducing their necessity or leveraging their presence. The eventual goal of such a system is to facilitate the natural functions of the trunk and allow neuroprosthesis users to maintain desired task-specific postures regardless of internal or external perturbations without requiring a support device.

## Data Availability

The datasets used and analyzed during the current study are available from the corresponding author on reasonable request.

## Abbreviations

SCI: Spinal cord injury
PID: Proportional integral derivative
ADL: Activities of daily living
FNS: functional neuromuscular stimulation
AIS: American Spinal Injury Association Impairment Score
ICC: Intraclass correlation coefficient
ES: Lumbar erector spinae
QL: Quadratus lumborum
PA: Posterior portion of adductor magnus
GX: Gluteus maximus
GM: Gluteus medius
IL: Iliopsoas
HS: Hamstring semimembranosus
IMU: Inertial measurement unit
